# Diagnostic Comparability and Interchangeability Between Daytime Ambulatory Blood Pressure Monitoring and 24-Hour Ambulatory Blood Pressure Monitoring in Detecting Masked Hypertension

**DOI:** 10.7759/cureus.11784

**Published:** 2020-11-30

**Authors:** Abdulhalim J Kinsara, Ahmed Abuosa, Alaa Meer, Aymen H Elsheikh, Mohammed Abrar, Olga Vriz

**Affiliations:** 1 Cardiology, Ministry of National Guard - Health Affairs, King Saud Bin Abdulaziz University for Health Sciences, College of Medicine, Western Region (COM-WR) - King Abdullah International Medical Research Center, Jeddah, SAU; 2 Cardiology, National Training Institute, Cairo, EGY; 3 Prince Noorah Oncology Center, Ministry of National Guard - Health Affairs, Jeddah, SAU; 4 Cardiology, King Faisal Specialist Hospital and Research Centre, Alfaisal University, Riyadh, SAU

**Keywords:** hypertension, daytime, ambulatory, blood pressure, masked, 24 h measurement, diagnosis, classification, agreement

## Abstract

Background

The primary aim of this study was to evaluate the level of diagnostic overlap between daytime ambulatory blood pressure (BP) monitoring (DT-ABPM) and 24-hour ambulatory BP monitoring (24-h ABPM) in detecting masked hypertension (MH).

Methods

This is a prospective study that was performed in a sample of 196 soldiers aged between 21 and 50 years (without a history of hypertension) undergoing ABPM testing. The diagnosis of MH based on DT-ABPM defined as (office blood pressure (OBP) <140/90 and DT-ABPM ≥135/85) was compared with the 24-h ABPM defined as (OBP <140/90 mm Hg and 24-h ABPM ≥130/80 mm Hg). We critically analyzed the results to see the agreement between the two methods.

Results

The number of subjects classified as having MH based on both DT-ABPM and 24-h ABPM, only on 24-h ABPM, and only on DT-ABPM were 11 (5.6%), 29 (14.8%), and 18 (9.2%), respectively. The sensitivity, specificity, and positive and negative predictive values for DT-ABPM in detecting MH were: sensitivity = 100% (95% CI: 97.82% - 100%), specificity = 62.07% (95% CI: 42.26% - 79.31%), PPV = 93.82% (95% CI: 90.50% - 96.03%), and NPV = 100%, respectively. The level of agreement between DT-ABPM and 24-h ABPM in diagnosing MH was 94.4% and discordance in 5.6% (11/196); (kappa=0.736, p < 0.001).

Conclusion

The sensitivity, specificity, positive and negative predictive values all showed agreement between the two BP methods to confirm the diagnoses of MH. DT-ABPM can be used as an alternative to the 24-h ABPM. DT-ABPM eliminates sleep disturbance attributable to ABPM and maximizes patient compliance with the ABPM test. A further larger trial is needed for more confirmation and to affect the guidelines for using daytime ABPM.

## Introduction

Ambulatory blood pressure monitoring (ABPM) is a non-invasive valuable technique for measuring and monitoring a person’s blood pressure (BP). It is considered the gold standard for the diagnosis of hypertension and assessment of cardiovascular risk [1-3]. The superiority of ABPM over conventional office blood pressure (OBP) in quantifying BP measurements for the diagnosing and management of hypertension is well-documented in the literature [4-6]. Some of the attractive features of ABPM include the following. First, it provides a profile of BP behavior over a 24-hour period or longer compared to a snapshot of BP taken in the doctor’s office. Second, the technique provides an accurate evaluation that reflects an individual’s true BP while performing during day-to-day activities and eliminates observer bias associated with OBP [7-8]. Third, ABPM is a precise technique for predicting cardiovascular events as compared to OBP measurements [9-12]. Fourth, the 24-h ABPM is the only technique that can accurately identify elevated nighttime BP and early morning surge [13]. Despite these advantages, the main drawback of 24-h ABPM is the sleep disturbance because of the device's frequent cuff inflation/deflation and noise during nighttime [14].

In the study by Viera et al., 70.2% of patients reported sleep disturbances, 19.6% unable to fall asleep, and 8.8% removed the ambulatory device during the night due to extreme sleep disturbance [6]. Recently, Ringrose et al. indicated that 78% of their patients experienced sleep disturbance caused by the monitoring device [15]. These findings suggest that the technique may cause repeated discomfort and sleep disturbance and hence may not be appropriate for every patient. In light of this, there is a need for a short, non-invasive BP measuring alternative technique to the 24-h ABPM method. DT-ABPM does not involve BP measurements during nighttime sleep and maybe an ideal diagnostic tool for MH. The recent guidelines from the European Society of Hypertension/European Society of Cardiology recommended the use of daytime, 24-hour, and/or nighttime to define MH [1]. Despite this, the overlap between DT-ABPM and 24-h ABPM in detecting MH remained an unexplored area of research.

The present study is a post hoc analysis of the database originally designed to compare the prevalence of MH as determined by DT-ABPM and 24-h ABPM [16]. However, the similarity of the prevalence rates does not necessarily mean the two BP methods are interchangeable in diagnosing/detecting MH. Thus, the primary objective of this post hoc analysis was to further assess the level of diagnostic overlap between DT-ABPM and 24-h ABPM in detecting MH (i.e. identify the identical individuals with MH and if we can accurately detect MH by DT-ABPM as a viable diagnostic alternative in the assessment and diagnosis of MH.

## Materials and methods

Study population

A total of 203 patients who attended the outpatient clinic over the last three years were recruited in this prospective study. Of these, 196 patients were eligible for analysis. The inclusion criteria were: male soldiers, age between 21 and 50 years, no previous history of hypertension or not taking antihypertensive medications, and OBP < 140/90 mmHg as previously described [16]. The local Institutional Review Board (IRB) approved this study. Participation in the study was voluntary and written informed consent was obtained from patients. Provisions for protecting privacy (access by unauthorized access) and/or confidentially were fully guaranteed.

Definition of masked hypertension

In the present study, the following definitions of MH have been adapted based on the European Society of Hypertension guidelines [1,17]. MH using 24-h ABPM was defined as patients with OBP of <140/90 mmHg and the 24-h ABP average ≥130/80 mmHg while MH using DT-ABPM is defined as patients with OBP of <140/90 mmHg and the daytime average blood ≥135/85 mmHg.

Office blood pressure

Patients were placed in an exam room. Prior to measuring their blood pressure, subjects were allowed to rest for a minimum of five minutes. OBP measurements were performed by trained nurses. All patients underwent two BP evaluations/measurements in the clinic. These BP measurements obtained in the sitting position were averaged to serve as a baseline for OBP and used in data analysis. Exclusion criteria were (1) age less than 21 or greater than 50 years; (2) OBP ≥ 140/90 mmHg; and (3) history of hypertension or antihypertensive medications. Patients with SBP > 180 mmHg were started on drug therapy while simultaneously scheduled for outpatient clinic follow-up visits. While patients with SBP > 140 but < 180 received lifestyle modification advice and scheduled to visit the outpatient clinic. Each patient who met the inclusion criteria was then fitted with an oscillometric device to undergo a 24-h ABPM. Patients were instructed to come back to the cardiac center the next day, 24 hours later, to remove the device.

Ambulatory blood pressure monitoring

The non-invasive ABPM was carried out using the oscillometric device, the same device used for both techniques (Tracker Reynolds, Reynolds Medical, UK). Participants were asked to adhere to their daily routine activities. The device was programmed to measure systolic and diastolic blood pressure values automatically every 30-minute intervals during daytime (between 6:00 AM to 10:00 PM) and every one hour during sleep/nighttime (between 10:00 PM to 6:00 AM). ABPM was considered adequate if at least 14 readings were recorded during the daytime. 

Following hypertension guidelines and applying cross-classification based on DT-ABPM and 24-h ABPM, patients were delineates into four groups: normotensive (patients with normal daytime); and three MH categories identified based on: both average DT-ABPM and 24-h ABPM (daytime ≥135/85 mmHg and 24-h ABP ≥130/80 mmHg); 24-h ABPM (≥130/80 mmHg); and DT-ABPM (≥135/85 mmHg).

Statistical analysis

Statistical analysis and estimates were performed using the Statistical Package for the Social Sciences (SPSS) software version 24.0 for Windows (IBM Corp, Armonk, NY). Categorical variables were evaluated by chi-square test and continuous BP values in the same participant were compared using the paired student's t-test. Using the 24-h ABPM method as the reference standard, the sensitivity (SN), specificity (SP), positive predictive value (PPV), and negative predictive value (NPV) were calculated. Correlation between BP readings was evaluated using the Pearson correlation coefficient. Measures of diagnostic performance: sensitivity, specificity, positive, and negative predictive values were computed. Cross-classification analysis and the Kappa coefficient were used to assess agreement between DT-ABP and 24-h ABP. A Kappa value of < 0.20 indicates poor agreement, 0.21-0.40 fair agreement, 0.41-0.60 moderate agreement, 0.61-0.80 substantial agreement, and 0.81-1.00 almost perfect agreement. Bland-Altman plots of the mean bias and 95% limits of agreements were generated to assess quantitative agreement between the two BP measurements. Statistical significance was set at p-value < 0.05.

## Results

**Figure 1:**
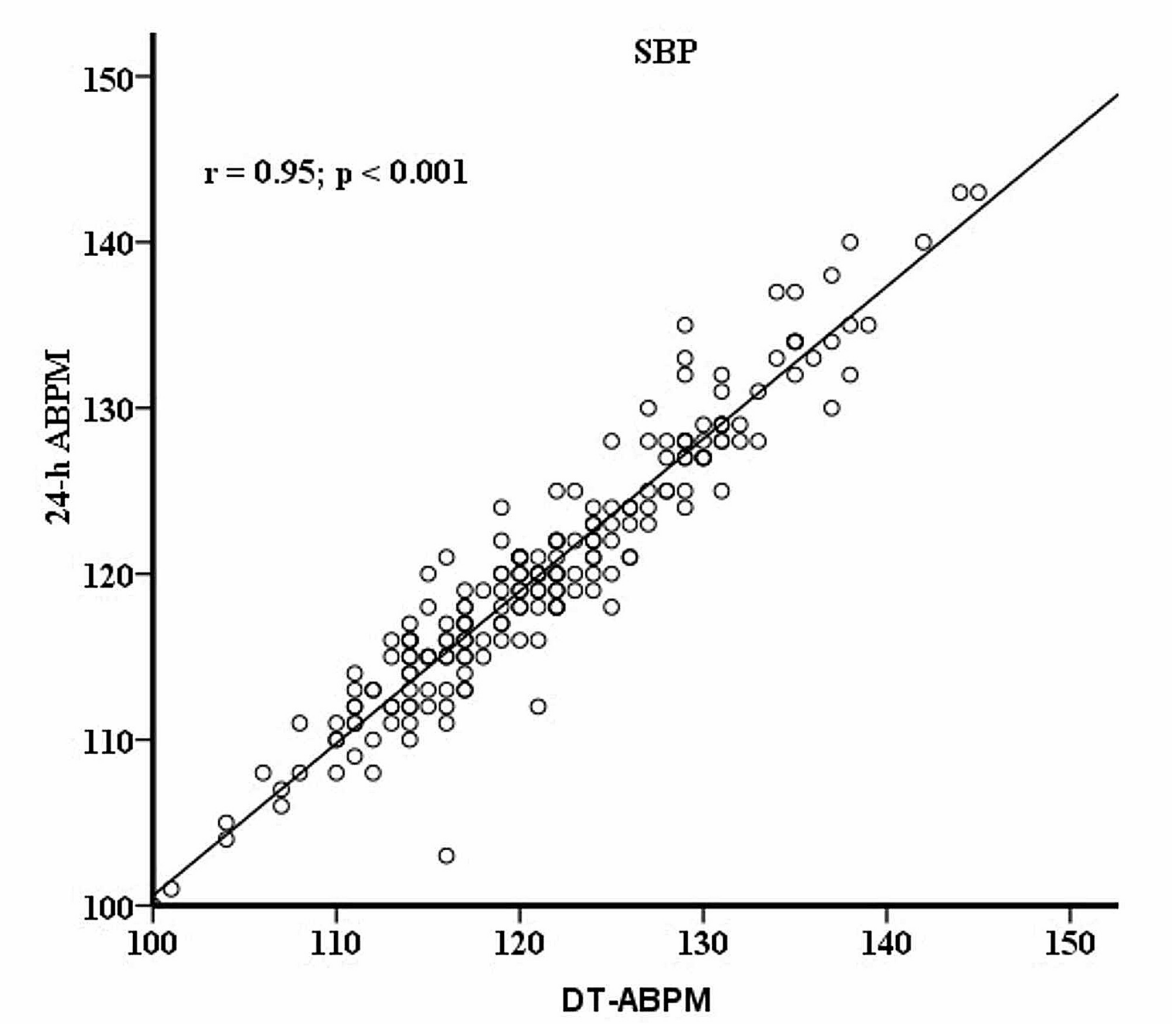
Systolic DT-ABPM and 24-h Correlation between systolic daytime-ambulatory blood pressure monitoring (DT-ABPM) and 24-hour ABPM r is the correlation coefficient.

In this study, 196 patients were analyzed. The mean age and standard deviation age were 32.7 and 5.3 years, respectively. The median age was 33 with a range: 21-50 years. Mean systolic and diastolic OBP were 121.5 +/-8.4 mmHg and 70.7+/- 7.0 mmHg, respectively. A strong and significant positive correlation between SBP readings was noted between DT-ABPM and 24-h ABPM (SBP: r = 0.952, p < 0.001) as depicted in Figure 1.

**Figure 2:**
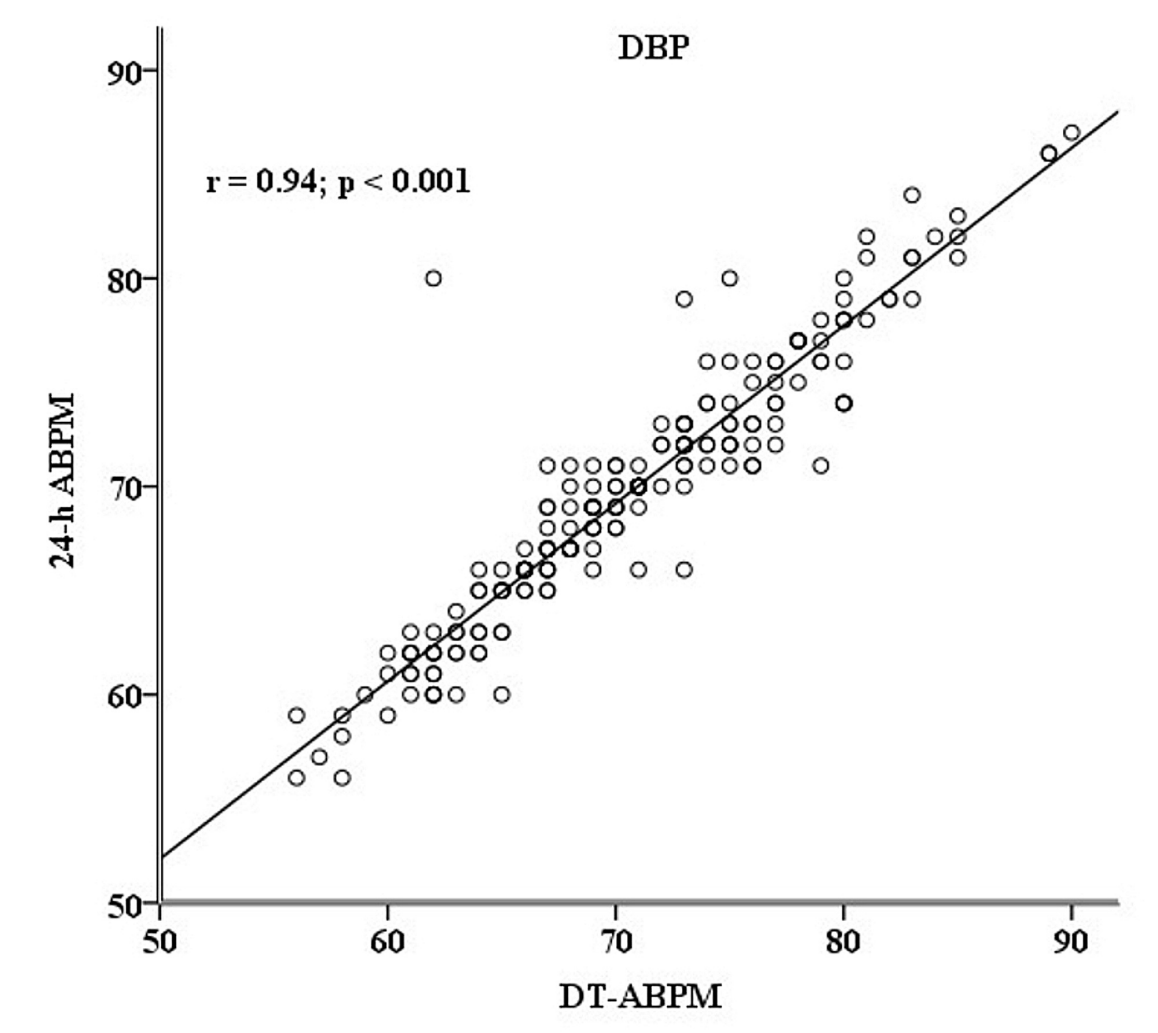
Diastolic DT-ABPM and 24-h ABPM Correlation between diastolic daytime-ambulatory blood pressure monitoring (DT-ABPM) and 24-hour ABPM

For diastolic blood pressure (DBP) measurement, the correlation was positive and of similar magnitude (DBP: r = 0.941, p < 0.001) as depicted in Figure 2.

**Figure 3:**
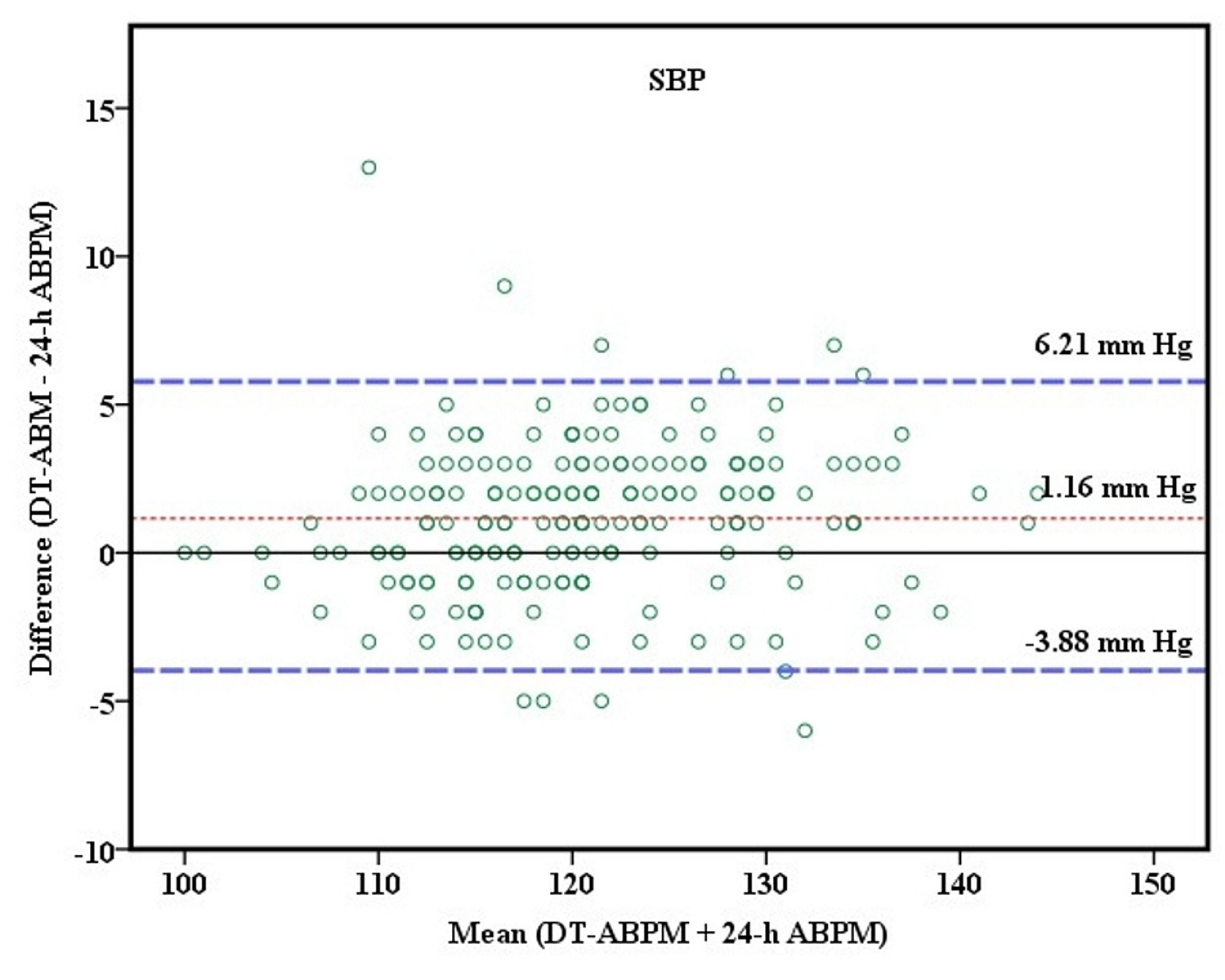
Agreement of systolic blood pressure between DT-ABPM and 24-h ABPM Bland-Altman plot showing agreement of systolic blood pressure between daytime-ambulatory blood pressure monitoring (DT-ABPM) and 24-h ABPM. Mean differences are shown in red dash-dot lines with ±2 SD in blue solid (dashed) lines.

The Bland-Altman graph was used to assess the degree of agreement between mean DT-ABPM and 24-h ABPM. The mean difference (bias) for SBP between the two methods was -1.16 mmHg and the standard deviation (SD) 2.57. The 95% limits of agreement were between −6.21 mmHg and 3.88 mmHg (Figure 3).

**Figure 4:**
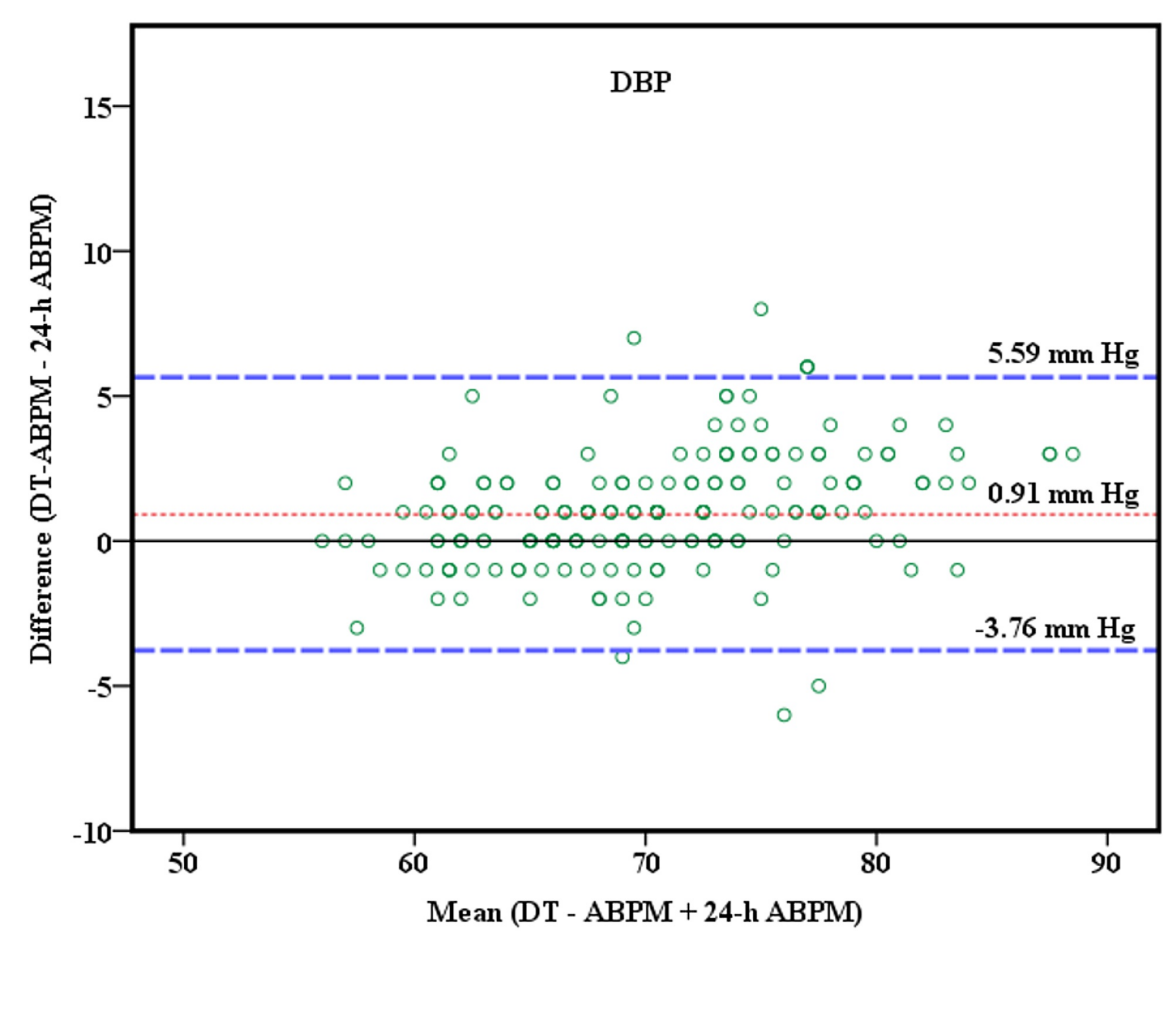
Agreement of Diastolic blood pressure between DT-ABPM and 24-h ABPM The Bland-Altman plot showing agreement of Diastolic blood pressure between daytime-ambulatory blood pressure monitoring (DT-ABPM) and 24-h ABPM. Mean differences are shown in red dash-dot lines with ±2 SD in blue solid (dashed) lines.

For DBP readings, the mean difference (bias) was -0.91 mmHg, and the standard deviation (SD) 2.38 with corresponding 95% limits of agreement −3.76 mmHg and 5.59 mmHg (Figure 4).

Among the 196 participants, 29 (14.8%) were classified as having MH using the 24-h ABPM and 18 (9.2%) using DT-ABPM (p = 0.119). Furthermore, 18 (9.2%) subjects were classified as having MH using both methods (DT-ABPM and 24-h ABPM), 11 (5.6%) using only 24-h ABPM, and none was detected using DT-ABPM alone. In total, 167 (85.2%) patients were classified as normotensive using both methods. In cases of diagnostic agreement, the same individuals were identified by the two methods and classified as hypertensive or normotensive.

The level of agreement between DT-ABPM and 24-h ABPM in detecting and classifying MH was 94.4% and discordance in 5.6% (11/196); (Kappa=0.736, p < 0.001). The sensitivity, specificity, and positive and negative predictive values for DT-ABPM in detecting MH were: sensitivity = 100% (95% CI: 97.82% - 100%), we wrote CI 0.59-0.88 in our paper, specificity = 62.07% (95% CI: 42.26% - 79.31%), PPV = 93.82% (95% CI: 90.50% - 96.03%), and NPV = 100%; respectively.

However, when a 5 mmHg gray zone of diagnostic uncertainty (i.e. clinically nonrelevant differences: <5 mmHg) was applied to the diagnostic thresholds, the estimated clinically important diagnostic disagreement between the two BP measurement methods was 1% and a near-perfect classification of 99.0% was achieved. The diagnostic disagreement was considered as clinically important in two of the 196 patients.

## Discussion

To the best of our knowledge, there are no studies to date that have investigated the overlap between DT-ABPM and 24-h ABPM for detecting MH in healthy subjects with an active lifestyle. The primary aim of the current study was to assess the level of diagnostic agreement between these BP methods in detecting/diagnosing MH. Our study revealed that DT-ABPM can accurately classify a patient’s MH status as determined by the 24-h ABPM. Furthermore, the study showed overwhelming diagnostic overlap between the two BP methods. The DT-ABPM method resulted in the same classification as the 24-h ABPM in 94.4% of the 196 cases and a substantial agreement (k = 0.736). Moreover, when a 5 mmHg gray zone for uncertain diagnosis was applied to the diagnostic threshold, the disagreement was reduced by less than 1% (a near-perfect classification of 99.0% was obtained). A similar finding was noted in a study of MH assessed using 24-h ABPM versus home BP monitoring (HBPM) conducted by Stergiou and colleagues [18].

The mean difference between the systolic DT-ABPM and systolic 24-h ABPM measurements was about 1 mmHg (Figure 3). A mean difference of similar magnitude was also noted between diastolic DT-ABPM and diastolic 24-h ABPM measurements (Figure 4). These findings imply the BP obtained by the two methods are similar and the difference is small with no clinical significance.

Our findings suggest that DT-ABPM is a viable alternative to the 24-h ABPM as a screening tool and in diagnosing MH in patients.

Accurate diagnosis is critically important in the treatment of hypertension. In the present study, the diagnostic agreement between DT-ABPM and 24-h ABPM (the gold standard) in detecting HT in untreated patients is striking high. Both BP methods eliminate observer bias and the white coat effect. However, DT-ABPM has a number of obvious advantages over 24-h ABPM: this technique eliminates sleep disturbance attributable to ABP monitoring, reduces patient discomfort, and maximizes patient acceptance [14-15]. Thus, DT-ABPM is a reliable alternative to the 24-h ABPM for patients who experience sleep disturbances and/or cannot endure performing the 24-h ABPM test in particular. Furthermore, since the two methods are interchangeable, DT-ABPM can serve as a gold standard instead of a 24-h ABPM to diagnose hypertension.

Strengths and limitations

Although both methods are the same in terms of the cost-effectiveness, ease of use, and safety and reusability of the device with almost identical diagnostic/classification accuracy, the DT-ABPM method is less time consuming, more convenient (short duration of the BP monitoring session) for patients, eliminates sleep disturbances, improves patient compliance with the BP monitoring, and, consequently, the sample size may be minimally affected. These features make DT-ABPM more practical and better suited for diagnosing MH than the 24-h-ABPM technique.

The current study has a few limitations. First, it is a single-center study. Second, the sample size is relatively small. Third, participants in the study are young men and not taking antihypertensive medication. As a result, our findings may not be generalized to the general public/larger population and need to be interpreted with caution. DT-ABPM lacks nighttime readings and does not capture nocturnal dipping patterns.

## Conclusions

This investigation adds important research evidence on ABPM. In clinical practice, the main drawback of 24-h ABPM is the sleep disturbance because of the device's frequent cuff inflation/deflation and noise during nighttime. The demonstration of non-inferiority of daytime ABPM to 24-hour ABPM has important clinical implications because it will facilitate wider use of this technique, allowing a more reliable diagnosis.

Further, larger randomized control trials are needed for more confirmation and to affect the guidelines for using daytime ABPM as an effective alternative for 24-hour ABPM. For the time being, home blood pressure monitoring could be used, but if daytime ABPM is confirmed by guidelines it will be more practical and helpful.
